# Gender-Dependent Effect of GSTM1 Genotype on Childhood Asthma Associated with Prenatal Tobacco Smoke Exposure

**DOI:** 10.1155/2014/769452

**Published:** 2014-09-18

**Authors:** Chih-Chiang Wu, Chia-Yu Ou, Jen-Chieh Chang, Te-Yao Hsu, Ho-Chang Kuo, Chieh-An Liu, Chih-Lu Wang, Chia-Ju Chuang, Hau Chuang, Hsiu-Mei Liang, Kuender D. Yang

**Affiliations:** ^1^Department of Pediatrics, Show Chwan Memorial Hospital, 542 Section 1, Zhongshan Road, Changhua 50008, Taiwan; ^2^Department of Medical Research, Show Chwan Medical System in Chang Bing, 6 Lu-Kung Road, Changhua 50544, Taiwan; ^3^Institute of Clinical Medicine, National Yang-Ming University, 155 Section 2, Linong Street, Taipei 11221, Taiwan; ^4^Department of Obstetrics and Gynecology, Kaohsiung Chang Gung Memorial Hospital, Taiwan and Chang Gung University College of Medicine, 123 Dapi Road, Kaohsiung 83301, Taiwan; ^5^Genomic and Proteomic Core Laboratory, Department of Medical Research, Kaohsiung Chang Gung Memorial Hospital and Chang Gung University College of Medicine, 123 Dapi Road, Kaohsiung 83301, Taiwan; ^6^Department of Pediatrics, Kaohsiung Chang Gung Memorial Hospital and Chang Gung University College of Medicine, 123 Dapi Road, Kaohsiung 83301, Taiwan; ^7^Department of Pediatrics, Po-Jen Hospital, 350 Bo'ai 2nd Road, Kaohsiung 81358, Taiwan

## Abstract

It remains unclear whether the GSTM1 genotype interacts with tobacco smoke exposure (TSE) in asthma development. This study aimed to investigate the interactions among GSTM1 genotype, gender, and prenatal TSE with regard to childhood asthma development. In a longitudinal birth cohort in Taiwan, 756 newborns completed a 6-year follow-up, and 591 children with DNA samples available for GSTM1 genotyping were included in the study, *and the interactive influences of gender-GSTM1 genotyping-prenatal TSE on childhood asthma development were analyzed*. Among these 591 children, 138 (23.4%) had *physician-diagnosed asthma* at 6 years of age, and 347 (58.7%) were *null-GSTM1*. Prenatal TSE significantly increased the prevalence of childhood asthma in *null-GSTM1* children relative to those with *positive* GSTM1. Further analysis showed that prenatal TSE significantly increased the risk of childhood asthma in girls with *null-GSTM1*. Furthermore, among the children without prenatal TSE, girls with *null-GSTM1* had a significantly lower risk of developing childhood asthma and a lower total IgE level at 6 years of age than those with *positive* GSTM1. This study demonstrates that the GSTM1 null genotype presents a protective effect against asthma development in girls, but the risk of asthma development increases significantly under prenatal TSE.

## 1. Introduction

The prevalence of childhood asthma has increased worldwide in recent decades [1]. Environmental factors, including increasing air pollution, tobacco smoke exposure, a lower load of infection with pathogens, increased use of industrial materials in buildings, urbanization, and certain nutritional factors, may play an important role in this evolving epidemic. Recently, increasing evidence has demonstrated that certain types of environmental exposure may increase the risk of asthma development for certain genetic backgrounds [2], implying that the gene-environment interaction is critical in asthma development.

Oxidative stress has been implicated in the pathogenesis of asthma, which is characterized by chronic airway inflammation. The glutathione S-transferases (GSTs) are a family of enzymes that have the general function of detoxifying xenobiotics that are capable of generating free radicals, by conjugating them with glutathione. GSTM1 has been extensively studied because its locus is polymorphic with a common null allele that produces a complete lack of the enzyme. The association between the GSTM1 null genotype and asthma development is not well established in the current literature. Several studies have demonstrated an increased risk of asthma or decreased lung function in subjects with the GSTM1 null genotype [3–9], whereas other studies have reported no association between the GSTM1 genotype and asthma [10–12]. The results of systematic reviews and meta-analyses of the effects of GSTM1 on asthma are also controversial. Some studies have revealed that the GSTM1 null genotype significantly increases the risk of asthma in children and adults [13, 14]. One meta-analysis showed that the GSTM1 null genotype may be associated with an increased risk of asthma (pooled OR 1.28; 95% CI 1.09–1.52), with large between-study heterogeneity. However, the association disappeared when the meta-analysis was repeated for the largest nine studies [15]. Another meta-analysis found no significant association between the GSTM1 polymorphism and asthma [16]. Several studies investigating the gene-environment interaction with regard to asthma development found that environmental oxidative stresses, such as tobacco smoke exposure [17–19] and ozone [20, 21], increased the risk of asthma in children with the GSTM1 null genotype but not in those with* positive* GSTM1. Another study showed that maternal use of acetaminophen in late pregnancy increased the risk of asthma or wheezing in children when the maternal or child's GSTM1 genotype was* positive* [22]. Functional studies on the role of GSTM1 in asthma are limited. Our previous studies have demonstrated that gene-gene and gene-environment interactions for IgE production begin in the prenatal stage [23–25]. This study aimed to investigate the effect of the GSTM1 genotype on the relationships among prenatal tobacco smoke exposure (TSE), childhood asthma development, and allergic sensitization for different gender backgrounds in a longitudinal birth cohort study in Southern Taiwan.

## 2. Methods

### 2.1. Study Design and Subjects

To study the effect of gene-gene and gene-environment interactions on prenatal and postnatal IgE production and the development of allergic diseases, a longitudinal birth cohort study was conducted at Kaohsiung Chang Gung Memorial Hospital, Taiwan, as reported previously [23–25]. In this cohort, the parents of 1848 children were prenatally recruited by our study nurse to enroll the birth cohort. Among the 1848 children, 1629 children were born in the hospital. In total, 1546, 1348, 1236, and 756 of the 1629 children completed the 6-month, 18-month, 3-year, and 6-year follow-up visits, respectively. DNA samples collected at the newborn stage (from the umbilical cord blood) and at 6 years of age were subjected to GSTM1 genotyping in this study. The study protocol was approved by the Institutional Review Board, and informed consent was provided to the parents at the prenatal stage. The information regarding parental atopy history and family smoking habits was obtained from a questionnaire administered during prenatal recruitment. We defined the infants as having prenatal TSE if any family member at home had a habit of smoking indoors at home. The atopy history of the children, including atopic dermatitis, allergic rhinitis, or asthma if ever diagnosed by a physician, was acquired from the questionnaire given to parents at the 6-year follow-up. Cord blood samples were collected immediately after the infant's birth for DNA collection. Blood samples were collected from children at the 6-year follow-up for DNA extraction and the measurement of allergen sensitization through the detection of specific IgE levels in response to egg whites (f1), cow's milk (f2), peanuts (f13), shrimp (f24), house dust mites (d1), and German cockroaches (i6) (Phadia CAP system) because house dust mites (approximately 90%) and German cockroaches, rather than pollen or mold (both <2%), are the major aeroallergens of children with asthma in Taiwan [26].

### 2.2. Analysis of GSTM1 Polymorphisms

Blood leukocytes were subjected to DNA extraction using the Gentra Puregene kit (Qiagen Inc., Valencia, CA) and then stored at −80°C after 70% alcohol precipitation.

The genetic polymorphism analysis for the GSTM1 genes was performed using an individual multiplex PCR approach [27] with the following primers: F 5′-CGCCATCTTGTGCTACATTGCCCG-3′ and R 5′-TTCTGGATTGTAGCAGATCA-3′ for GSTM1 and F 5′-CAACTTCATCCACGTTCACC-3′ and R 5′-GAAGAGCCAAGGACAGGTAC-3′ for *β*-globin. Briefly, the multiplex PCR was performed in a 25 *μ*L reaction mixture consisting of 50 ng of DNA, 2.5 *μ*L of 10× GenTaq buffer, 10 mM dNTP mix, each primer at 10 *μ*M, and 1 U of GenTaq DNA polymerase. The PCR reaction was performed in a PCR thermal cycler system (Applied Biosystems, Life Technologies) with an initial denaturation at 95°C for 5 min and then 35 cycles of 50 s at 94°C, 50 s at 60°C, and 50 s at 72°C, followed by a final elongation for 10 min at 72°C. The amplified products were visualized on a 2% agarose gel. The GSTM1 genotypes were determined by the presence or absence of bands at 230 bp, with a 260 bp internal control (*β*-globin). Through this simple multiplex PCR approach, the null and* positive* genotypes for GSTM1 could be clearly identified, although heterozygous and homozygous positive genotypes could not be differentiated.

### 2.3. Data Analysis and Statistics

The demographic data for the children with or without GSTM1 genotypes and the prevalence of allergic diseases and allergic sensitization at 6 years of age in children with or without prenatal TSE were analyzed using the chi-squared test. Multiple logistic regressive analyses were performed for childhood asthma with several factors, including preterm, gender, paternal and maternal atopy, and prenatal TSE. The log-transformed IgE levels at 6 years of age among the groups were analyzed by one-way ANOVA and Bonferroni post hoc tests.

## 3. Results

### 3.1. Demographic Data for the Birth Cohort with 6-Year Follow-Up

Of the cohort of 756 children who completed the follow-up at 6 years of age, 591 subjects, whose DNA samples collected as newborns and at 6 years of age were available, were subjected to GSTM1 genotyping in this study. Among these 591 subjects, 244 (41.3%) were* positive* and 347 (58.7%) were null for the GSTM1 genotype. In total, 188 (31.8%), 320 (54.1%), and 138 (23.4%) of these children had been diagnosed at 6 years of age with atopic dermatitis, rhinitis, or asthma, respectively, by a physician. In total, 579 of the 591 children received an allergic sensitization test at the 6-year follow-up, and 256 (43.3%) children were sensitized to house dust mites. Among the 591 children analyzed in this study, children with prenatal TSE had a significantly higher risk of developing asthma than children without prenatal TSE (*P* = 0.006, OR: 1.780, 95% CI: 1.181–2.684). However, there were no significant differences in the development of atopic dermatitis or rhinitis between children with and without prenatal TSE (Table 1). Moreover, there were no differences in food allergen sensitization or house dust mite sensitization between these 2 groups (data not shown).

### 3.2. Prenatal TSE Increases the Risk of Childhood Asthma in Null-GSTM1 Subjects Compared to* Positive* GSTM1 Children

Next, the relationship between prenatal TSE and childhood asthma in children with the GSTM1 null or* positive* genotype was analyzed. Prenatal TSE significantly increased the prevalence of childhood asthma in children with the GSTM1 null genotype (*P* = 0.002, OR: 2.337, 95% CI: 1.370–3.985) compared with those with* positive* GSTM1 (*P* = 0.550) (Table 1). Furthermore, a multivariate logistic regression analysis of childhood asthma in children with the GSTM1 null or* positive* genotype was performed to adjust for other demographic data, such as gender, prematurity, and maternal and paternal atopy. In the multivariate logistic regression analysis, male gender and prenatal TSE both significantly increased the prevalence of childhood asthma in children with the GSTM1 null genotype, whereas prenatal TSE was not a significant risk factor in children with* positive* GSTM1 (Table 2).

### 3.3. Gender-Dependent Association between GSTM1 Null Genotype and Asthma

**Figure 1:**
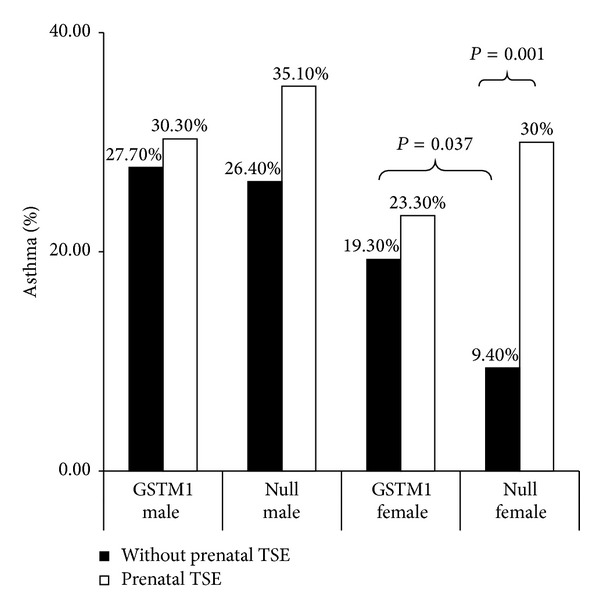
Interactions among GSTM1 genotype, gender, and prenatal TSE with regard to asthma development at 6 years of age. Prenatal TSE significantly increased the risk of childhood asthma in females with the GSTM1 null genotype (*P* = 0.001, OR: 4.107, 95% CI: 1.669–10.107), but not in the other 3 groups. Furthermore, among children without prenatal TSE, females with the GSTM1 null genotype had a significantly lower risk of developing childhood asthma (*P* = 0.036, OR: 0.436, 95% CI: 0.197–0.966).

To analyze the association between prenatal TSE and childhood asthma for the GSTM1 null and* positive* genotypes and in different gender backgrounds, we categorized all of the children into four groups (males with* positive* GSTM1 genotype, males with GSTM1 null genotype, females with* positive* GSTM1 genotype, and females with GSTM1 null genotype). Prenatal TSE significantly increased the risk of childhood asthma in females with the GSTM1 null genotype (30.0% versus 9.4%, *P* = 0.001, OR: 4.107, 95% CI: 1.699–10.107) compared with the effect in the other 3 groups (Table 3 and Figure 1). Furthermore, among children without prenatal TSE, females with the GSTM1 null genotype had a significantly lower risk of developing childhood asthma compared with females with* positive* GSTM1 (9.4% versus 19.3%, *P* = 0.037, OR: 0.436, 95% CI: 0.197–0.966) (Table 3 and Figure 1).

### 3.4. Gender-Dependent Association between GSTM1 Null Genotype and Total IgE Levels

**Figure 2:**
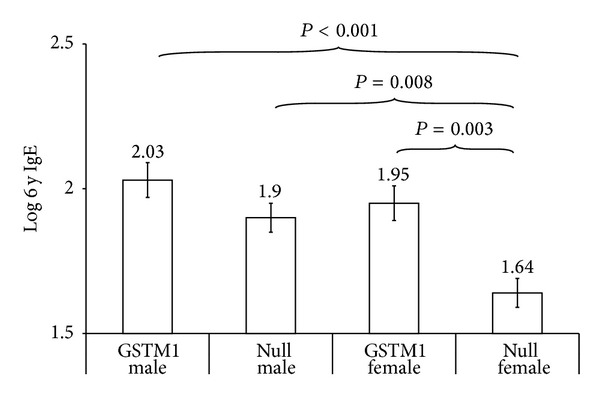
Interaction of the GSTM1 genotype and gender with regard to the log-transformed total IgE level at 6 years of age among individuals without prenatal TSE. Among children without prenatal TSE, the log-transformed total IgE level at 6 years of age was significantly lower in females with the GSTM1 null genotype than in the other 3 groups. The results are presented as the mean ± standard error.

Among the children without prenatal TSE, the log-transformed total IgE level at 6 years of age was significantly lower in female children with the GSTM1 null genotype than in the other 3 groups (Figure 2). Additionally, male and female children with the GSTM1 null genotype presented lower house dust mite and food allergen sensitization compared with those with* positive* GSTM1, but the difference was not significant (Table 4).

## 4. Discussion

This study demonstrates that the GSTM1 null genotype could have a bipolar effect on childhood asthma development, depending on gender and prenatal TSE. In particular, the GSTM1 null genotype is a protective factor against asthma in girls without prenatal TSE but becomes a risk factor for asthma with prenatal TSE.

Asthma is a chronic inflammatory airway disorder associated with airway hyperresponsiveness and reversible airflow limitation in response to specific triggers. Disturbances in oxidation/reduction (redox) reactions and impaired antioxidant defenses have been associated with asthma [28], and lower systemic GSH levels and higher GSSG levels have been reported to be related to asthma development and increased asthma severity [29–31]. GSTM1, one of the most studied enzymes in the GST family, functions in the detoxification of xenobiotics, environmental toxins, and products of oxidative stress by conjugation with glutathione, thereby protecting cells from reactive oxygen species (ROS). Individuals with the GSTM1 null genotype lose GSTM1 enzymatic activity and thus may be vulnerable to oxidative stress in the airway and have a higher risk for airway inflammation. Most previous studies have reported that the GSTM1 null genotype significantly increases the risk for asthma development, although some studies found this risk to be insignificant. Wu et al. found that stimulation of primary human bronchial epithelial cells (*positive* GSTM1) with diesel exhaust particles (DEP) significantly increased IL-8 and IL-1*β* protein expression, and knockdown of GSTM1 in these cells further elevated DEP-induced IL-8 and IL-1*β* expression, implying that GSTM1 deficiency aggravates DEP-induced proinflammatory responses [32].

Similar to a previous study showing that the GSTM1 null genotype increases the risk of childhood asthma only in children with a history of intrauterine smoke exposure [33], this study found that the GSTM1 null genotype is associated with a higher incidence of asthma than the* positive* GSTM1 genotype is in boys and girls with prenatal TSE, with a nonsignificant difference (35.3% versus 30.3%, *P* = 0.643, OR: 1.243, 95% CI: 0.495–3.121 for boys, 30.0% versus 23.3%, *P* = 0.535, OR: 1.408, 95% CI: 0.477–4.159 for girls). Additionally, we found that prenatal TSE increases the risk of childhood asthma in individuals with the GSTM1 null genotype, which supports the findings of other studies [17–19]. Furthermore, we found that prenatal TSE significantly increases the risk of childhood asthma in girls with the GSTM1 null genotype, but not in boys with the GSTM1 null genotype.

Among the children without prenatal TSE, girls with the GSTM1 null genotype had a significantly lower incidence of asthma and lower log-transformed IgE level at 6 years of age than girls with* positive* GSTM1. Additionally, among individuals (either boys or girls) without prenatal TSE, the GSTM1 null genotype was associated with lower house dust mite or food allergen sensitization, but the difference was not significant. These results suggest that the GSTM1 null genotype may protect against the development of asthma in girls if no prenatal TSE is present. This hypothesis is supported by a study in which the GSTM1 null genotype is associated with a decreased risk for asthma among atopic subjects [34] and another study investigating the effect of the* positive* and null-GSTM1 genotypes on allergen-induced oxidant stress, airway inflammation, and reactivity in vivo in adults with mild atopic asthma and without a regular asthma medication [35]. In this study, patients with a* positive* genotype had higher baseline and allergen-provoked airway neutrophilia and higher concentrations of myeloperoxidase than GSTM1 null patients. The allergen-stimulated generation of the acute-stress and proneutrophilic mediators tumor necrosis factor-α, CXCL-8, IL-1*β*, and IL-6 and the postallergen airway concentrations of IgE and the neutrophil-generated mediators matrix metalloproteinase-9, B-cell activating factor, transforming growth factor-*β*1, and elastase were also higher in patients with* positive* GSTM1. This study also found that GSTM1* positive* individuals with asthma were more reactive to specific allergens, producing a 20% decrease in FEV1, but eosinophil inflammation and allergen-induced F2-isoprostane levels, which are considered to be specific markers of oxidative stress in vivo, were unaffected. These findings imply that certain phenotypes of asthma may be affected by the GSTM1 genotype.

There are certain limitations in this study. Not all children who completed the 6-year follow-up had DNA samples qualified for GSTM1 genotyping, making selection bias possible. However, the demographic data on the 591 and 165 children with and without available DNA samples, respectively, including data on parental allergic diseases and sensitization, gender, prematurity, and the presence or absence of prenatal TSE, were not significantly different (data not shown). Moreover, our study examined TSE at the prenatal stage but not in the infant stage or childhood stage.* Furthermore, we acknowledge that caution must be used in generalizing these results to other populations, because the GST loci strongly interact with the environment. The different environmental risk factors, which are present in human populations, may certainly influence the results of this type of genetic association studies*.

This study yields additional evidence for the gene-environment interaction with regard to the development of childhood asthma and IgE level, starting in the prenatal stage. Based on the present findings, the GSTM1 null genotype in girls may be a protective factor against childhood asthma development and IgE level. However, girls with the GSTM1 null genotype are much more vulnerable to environmental oxidative stresses, such as prenatal TSE. The mechanism may not involve simple redox equilibrium because the GSTM1 genotype (null or* positive*) was previously found to be unrelated to the total plasma GST enzymatic activity, and the lack of GSTM1 activity for the GSTM1 null genotype may be compensated by other members of the GST superfamily [36]. Similarly, another study also found that* positive* GSTM1 was possibly related to neutrophilic inflammation in asthma without affecting allergen-induced F2-isoprostane levels [35]. Further studies are necessary to investigate the exact mechanism by which the GSTM1 genotype influences airway inflammation and allergic responses.

## 5. Practical Implications

This study demonstrates a gene-environment-gender interaction in the development of childhood asthma. Specifically, prenatal TSE increases the prevalence of childhood asthma. The GSTM1 null genotype is a protective factor against asthma development in girls without prenatal TSE but becomes a risk factor with prenatal TSE.

## Figures and Tables

**Table 1:** Allergic diseases in children with or without prenatal TSE at 6 years of age (6 y) and with different GSTM1 genotypes.

	Prenatal TSE	No prenatal TSE	*P*	OR	95% CI
6 y dermatitis	48/156	140/435	0.745	0.937	0.631–1.390
GSTM1 null	30/95	77/252	0.854	1.049	0.631–1.745
GSTM1 *positive*	18/61	63/183	0.480	0.797	0.425–1.496

6 y rhinitis	90/156	230/435	0.300	1.215	0.840–1.758
GSTM1 null	60/95	134/252	0.095	1.510	0.930–2.451
GSTM1 *positive*	30/61	96/183	0.657	0.877	0.491–1.566

6 y asthma	49/156	89/435	0.006	1.780	1.181–2.684
GSTM1 null	32/95	45/252	0.002	2.337	1.370–3.985
GSTM1 *positive*	17/61	44/183	0.550	1.221	0.634–2.348

**Table 2:** Multivariate regression analysis of childhood asthma in children with the GSTM1 null or *positive* genotype.

	GSTM1 null			GSTM1 *positive*		
	*P*	OR	95% CI	*P*	OR	95% CI
Male gender	0.003	2.317	1.342–4.000	0.126	1.606	0.875–2.950
Preterm (<37 weeks)	0.583	0.725	0.230–2.285	0.723	0.784	0.205–2.999
Maternal atopy^†^	0.488	1.235	0.681–2.240	0.595	1.206	0.604–2.409
Paternal atopy^†^	0.228	1.419	0.803–2.509	0.125	1.643	0.871–3.101
Prenatal TSE	0.003	2.308	1.333–3.997	0.553	1.223	0.629–2.377

^†^Atopy is defined by phenotypic asthma, allergic rhinitis, or atopic dermatitis along with a detectable serum specific IgE (≥0.35 kU/L) response to one or more common allergens (egg white, cow's milk, peanut, shrimp, house dust mite, or German cockroach).

**Table 3:** Association of prenatal TSE and childhood asthma in individuals of different genders and GSTM1 genotypes.

		Childhood asthma				
		No TSE Asthma number/total number (percentage)	Prenatal TSE Asthma number/total number (percentage)	*P*	OR	95% CI
		OR (95% CI)	OR (95% CI)			
Male	GSTM1	28/101 (27.7%)	10/33 (30.3%)	0.775	1.134	0.479–2.681
		1.000 (reference)	1.000 (reference)			
Male	Null	32/129 (26.4%)	20/57 (35.1%)	0.227	1.510	0.773–2.952
		0.933 (0.519–1.676)	1.243 (0.495–3.121)			

Female	GSTM1	17/88 (19.3%)	7/30 (23.3%)	0.637	1.271	0.469–3.448
		1.000 (reference)	1.000 (reference)			
Female	Null	12/127 (9.4%)	12/40 (30.0%)	0.001	4.107	1.669–10.107
		0.436 (0.197–0.966)	1.408 (0.477–4.159)			

**Table 4:** The GSTM1 null genotype presented a lower prevalence of house dust mite sensitization and food allergen sensitization compared with *positive* GSTM1 in boys and girls without prenatal TSE, but the difference was not significant.

	Male					Female				
	GSTM1		Null		*P*	GSTM1		Null		*P*
6 y HDM sensitization∗	51/100	51.0%	57/126	45.2%	0.389	37/87	42.5%	48/122	39.3%	0.644
6 y food allergen sensitization^#^	27/100	27.0%	31/126	24.6%	0.682	22/87	25.3%	23/122	18.9%	0.265
6 y any sensitization^†^	59/100	59.0%	66/126	52.4%	0.32	42/87	48.3%	52/122	42.6%	0.418

*HDM sensitization is defined by a specific IgE response to house dust mites (d1) ≧0.35 kU/L.

^#^Food allergen sensitization is defined by a detectable serum specific IgE (≥0.35 kU/L) response to one or more food allergens (egg white, cow's milk, peanut, or shrimp).

^†^Any sensitization is defined by a detectable serum specific IgE (≥0.35 kU/L) response to one or more common allergens (egg white, cow's milk, peanut, shrimp, house dust mite, or German cockroach).
